# Transfer learning strategies for solar power forecasting under data scarcity

**DOI:** 10.1038/s41598-022-18516-x

**Published:** 2022-08-27

**Authors:** Elissaios Sarmas, Nikos Dimitropoulos, Vangelis Marinakis, Zoi Mylona, Haris Doukas

**Affiliations:** 1 Decision Support Systems Laboratory, School of Electrical and Computer Engineering, National Technical University of Athens, 15780 Athens, Greece; 2 HOLISTIC IKE, 15343 Athens, Greece

**Keywords:** Solar cells, Electrical and electronic engineering, Computer science

## Abstract

Accurately forecasting solar plants production is critical for balancing supply and demand and for scheduling distribution networks operation in the context of inclusive smart cities and energy communities. However, the problem becomes more demanding, when there is insufficient amount of data to adequately train forecasting models, due to plants being recently installed or because of lack of smart-meters. Transfer learning (TL) offers the capability of transferring knowledge from the source domain to different target domains to resolve related problems. This study uses the stacked Long Short-Term Memory (LSTM) model with three TL strategies to provide accurate solar plant production forecasts. TL is exploited both for weight initialization of the LSTM model and for feature extraction, using different freezing approaches. The presented TL strategies are compared to the conventional non-TL model, as well as to the smart persistence model, at forecasting the hourly production of 6 solar plants. Results indicate that TL models significantly outperform the conventional one, achieving 12.6% accuracy improvement in terms of RMSE and 16.3% in terms of forecast skill index with 1 year of training data. The gap between the two approaches becomes even bigger when fewer training data are available (especially in the case of a 3-month training set), breaking new ground in power production forecasting of newly installed solar plants and rendering TL a reliable tool in the hands of self-producers towards the ultimate goal of energy balancing and demand response management from an early stage.

## Introduction

Urbanization effects are obvious during the last decades, as more and more people move to cities, resulting in more than 90% of anthropogenic carbon emissions being generated in urban environments^1^. This new reality has highlighted the need for a gradual transition to smart cities, where operational efficiency is improved with the use of emerging information and communication technology (ICT) applications^2,3^. At the same time, the impact of the digitalization era is more evident than ever, improving quality of life through digital automation of complex processes^4,5^. This transformation could not leave the energy sector unaffected, as the convergence of electrical power and data offers opportunities for new services^6^, with the aim of reducing costs and reshaping business models^7^. In the context of an ever-evolving urban environment, it is important to promote the concept of energy communities; i.e., the organization of collective energy actions around open, democratic participation and governance^8^.

A major point of interest for energy communities is effectively forecasting electricity production of Renewable Energy Sources (RES), as it is vital for balancing electricity supply and demand, and consequently, for scheduling and analyzing distribution networks and ensuring community autarky^9^. More specifically, rooftop photovoltaic systems are one of the most promising energy generation sources for prosumers in big cities, as more and more PV panels are installed^10^. Studies have shown that rooftop PVs can cover almost completely the domestic electricity demand for prosumers, indicating that self-sufficient cities are expected to be protagonists of the energy transition era^11^. However, efficient prosumption schemes are based on accurate solar production forecasting models. The problem becomes more demanding when lack of data is taken into account, which is a common phenomenon in the case of newly installed photovoltaic systems, where it takes a long time to collect a sufficient sample of data. This paper aspires to present a Transfer Learning (TL) approach for PV production forecasting in the case of lack of data, where predictive Deep Learning (DL) models are trained in PV plants with data adequacy, and transfer knowledge to PV plants with a small sample of available data.

Several approaches have been proposed for the problem of energy production forecasting in PV plants. The simplest approach is the naive persistence method, which assumes that the generated power will remain the same as the previous observation. A more complex variation of this approach is the smart persistence method, which additionally assumes that the clear-sky index^12,13^ of power output is the same in the future as in now. These approaches are usually used as reference models against other more complex methods. Apart from persistence methods, many data-driven methods have been proposed including time series forecasting models, spatio-temporal statistics, and machine learning (ML) techniques. According to Bacher et al.^14^, there are two dominant approaches for solar power forecasting: The first approach requires that solar power is normalized with a clear sky model in order to formulate a more stationary time series facilitating forecasting with classical linear time series methods. The second approach includes utilization of neural networks (NNs) with different types of input to predict the solar power directly. Traditional statistical approaches and time series models have been utilized to a great extent for short-term and long-term predictions. Indicative examples in this category are the Autoregressive Moving Average (ARMA) model proposed by Huang et al.^15^, a series of statistical regression methods presented by Zamo et al.^16^ and exponential smoothing for solar irradiance forecasting developed by Dong et al.^17^. Except from statistical models, the problem of PV-based power forecasting has been successfully tackled by exploiting ML methods. Studies based on Support Vector Machine (SVM) models^18^, the k-nearest neighbors algorithm^19^ and tree based models, such as Random Forest^20^ (RF) and Decision Trees^21^, are indicative examples of how efficient ML methods can be for this problem. However, the aforementioned approaches have been outperformed by DL models which have emerged in the last decade^22^. The increased processing power afforded by graphical processing units (GPUs) has resulted in the rise of DL, offering the opportunity to solve a wide range of problems with good accuracy^23^. More specifically, Feedforward Neural Networks^24^ (FFNN), Recurrent Neural Network (RNN) models, as well as their most recent variation called Long Short-Term Memory^25^ (LSTM) models, have gained ground over traditional statistical and ML methods regarding the PV production forecasting problem. In addition, a widely adopted category of methods are the so-called hybrid methods, which are essentially a combination of ML or DL models, either in the form of ensembles^26^ or using more elaborate methods such as boosting, bagging or meta-learning^27^. In this paper, a stacked LSTM architecture, i.e., an LSTM model comprised of multiple LSTM layers, is selected for two reasons. Firstly, LSTM can represent the dynamic performance of systems, being capable of efficiently handling sequential data with temporal relationships. Such temporal, non-linear, relationships exist between weather variables and the PV power output, constituting LSTM as an appropriate model for this forecasting problem. Secondly, being a weight-based model, unlike other statistical methods, it is suitable for the application of TL approaches.

However, DL models are data consuming, in general, requiring a sufficient amount of data in order to achieve high accuracy predictions. Even more interestingly, DL models are more data dependent than traditional time series forecasting techniques and ML models, which is compensated by their increased predictive capability. In this context, it is generally acknowledged that DL models trained with too little data suffer from under-fitting and that they result in poor approximation and, consequently, in high variance estimation of the model’s performance. The cause of this problem is known as data scarcity. More specifically, data scarcity can be defined as the situation where there is a limited amount of training data. In general, there are two different forms of data scarcity when dealing with PV power output data. First and foremost, data scarcity is a common phenomenon in the case of newly installed photovoltaic systems, where it takes a long time to collect a sufficient sample of power output data in order to train the models. Secondly, data scarcity may be attributed to missing values (or data gaps) due to malfunctioning smart-meters. In either case, the result is a lack of data to train a DL forecasting model from scratch. In the case of PV production forecasting a sufficient amount of training data is one calendar year, in order to enable the model to learn seasonal patterns. In order to tackle this problem, we exploit a model which is trained for one location and we apply it to another location where there is too little historical data.

Humans have the innate ability of exploiting information collected from one task in order to resolve similar tasks. The same applies in the field of DL^28^. Traditional DL approaches rely on learning new concepts from scratch, using data from a specific topic in order to train the model. However, the exploitation of data from similar applications can facilitate the learning process. TL has been proven to be very efficient for dealing with problems with insufficient or missing data. Data scarcity often occurs due to data being inaccessible, high cost of data collection mechanisms and Internet of Things (IoT) devices, or lack of appropriate data storage schemes. However, the impact of the digitisation era is more apparent than ever, as data availability and data quality have significantly improved. Thus, big data repositories enable the exploitation of existing datasets in order to address similar problems, rendering TL the most suitable approach^29^. Focusing on the energy sector, TL emerges as the most popular technique for problems with insufficient or poor-quality data. A typical example is the Hephaestus method for cross-building energy forecasting, considering seasonality and trend factors^30^. Similar studies have been developed for short-term building energy predictions^31^ and energy consumption forecasting with poor-quality data^32^. However, few studies have addressed the problem of PV production forecasting with TL^33^, allowing room for further research in this area.

In this paper, we explore three different TL strategies and we evaluate the impact of TL in providing accurate PV production forecasts, comparing the efficiency of traditional and TL models with respect to data availability. Results indicate that TL models significantly outperform the conventional one, achieving 24.8% accuracy improvement with 1 year of training data. The gap between the methods is even bigger when fewer training data are available (3-month training set), breaking new ground in power production forecasting of newly installed solar plants and rendering TL a reliable tool in the hands of self-producers towards the ultimate goal of energy balancing and demand response management from an early stage.

The rest of the paper is organized as follows. The second section (Methods) introduces the experimental data and processing, the TL models and proposed architectures. The results of this study are presented in the third section (Results), describing the baseline model performance, the validation process of the proposed TL strategies, and the data availability impact. Finally, the fourth section (Discussion) summarizes the conclusion.

## Methods

### Experimental data and processing

Hourly PV production and weather data (temperature, humidity and solar irradiance) from 7 PV plants are exploited. PV production data are collected directly from the solar plant systems of a Portuguese energy community, while weather data are extracted from a local meteorological station^34^ and the Copernicus Atmosphere Data Store^35^. One PV plant, namely $PV_1$, is used as the base model and the other PV plants are used for the development of the TL models. Specific information for the examined PV plants are presented in Table 1.

**Table 1:** Information of the PV plants that were used for the experimental application of the study.

PV	Location	Latitude	Longitude	Nominal(kW)	Peak(kW)	Avail. Data (rows)	From date	To date
$PV_1 (base\ PV)$	Lisbon	38.728	$-$ 9.138	23.52	20.00	21956	01/08/2018	31/01/2021
$PV_2$	Lisbon	38.833	$-$ 9.191	46.00	40.00	21908	31/12/2018	01/01/2021
$PV_3$	Setubal	38.577	$-$ 8.872	271.53	216.00	9670	02/02/2020	10/03/2021
$PV_4$	Lisbon	38.725	$-$ 9.120	30.00	27.00	20588	01/08/2018	31/01/2021
$PV_5$	Faro	37.031	$-$ 7.893	60.48	50.00	15044	08/09/2019	30/04/2021
$PV_6$	Braga	41.493	$-$ 8.496	119.88	108.00	18045	10/01/2019	31/01/2021
$PV_7$	Lisbon	38.701	$-$ 9.236	55.65	50.00	21932	01/08/2019	31/01/2021

**Figure 1:**
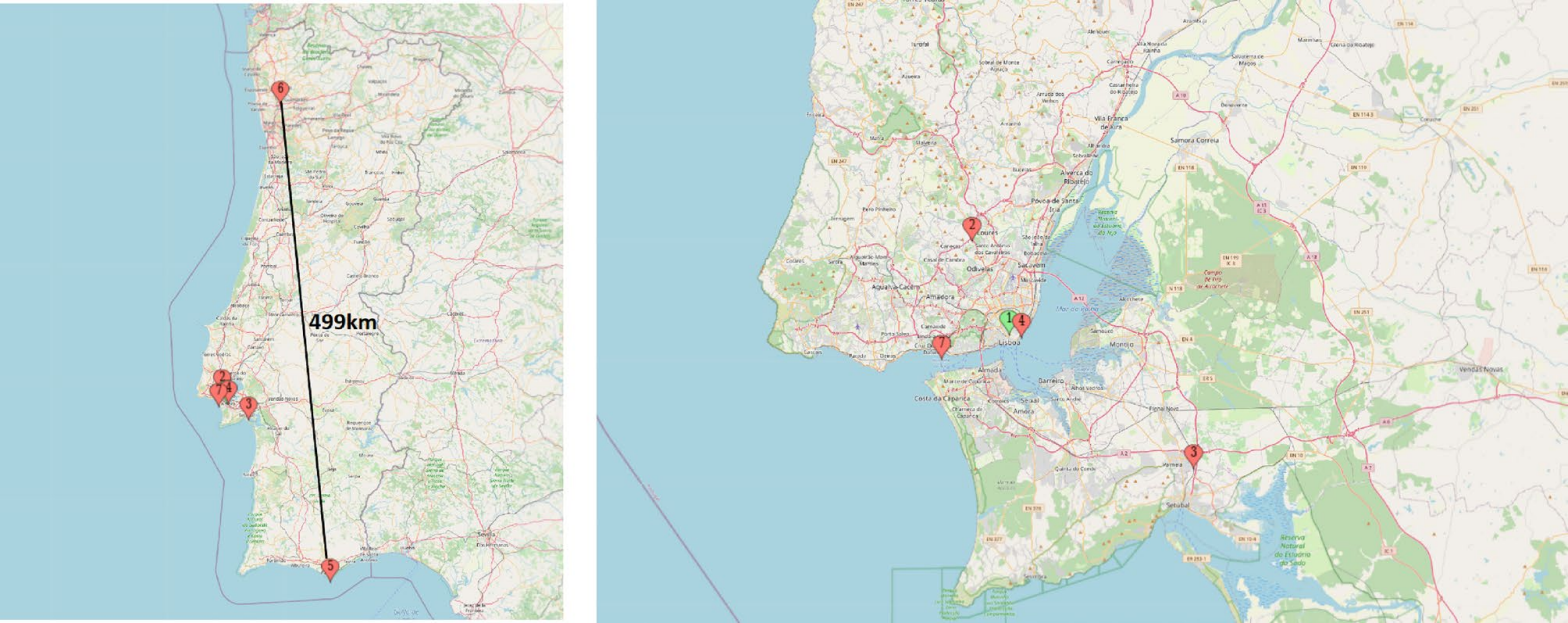
A map depicting the locations of the inspected PV plants. The 7 PV plants are located in four different Portuguese cities (Lisbon, Setubal, Faro, Braga) allowing for assessment of model performance regardless of the city. The image on the right offers a focus on the city of Lisbon where 4 PV plants are located (map created with https://www.mapcustomizer.com/, OpenStreetMap contributors).

The PV plants are located in 4 cities in Portugal (4 PVs are located in Lisbon; 1 is located in Setubal, Faro and Braga, respectively) and the available data vary from 14 to 30 months depending on the PV plant. The selected PV plants also differ in terms of nominal and peak capacities. The base PV has a nominal capacity of 23.52 KW, while the target domain PVs’ capacity varies from 30 KW to 271.53 KW, which is over 10 times the capacity of the base PV. The rationale behind the selection of the specific PV plants is to assess model performance on PV plants that are located both in the same city ($PV_2$, $PV_4$ and $PV_7$) and in different cities to the base PV ($PV_3$, $PV_5$ and $PV_6$), in order to report potential differences in the TL models’ forecasting accuracy. The locations of the inspected PV plants are depicted in Fig. 1.

The selected features of the stacked LSTM model are temperature, humidity, solar irradiance, PV production, one-hot encoding representation of the month of the year and sine/cosine transformation of the hour of day. The following processing routine is conducted for each dataset: Firstly, data are normalized to [0, 1] range. Secondly, data are transformed to “5 inputs - 1 output” format to be processed by the stacked LSTM model. Thirdly, the datasets are split into train sets and test sets. The base model is trained on the whole dataset of the source domain. The TL models are trained on 12 months of data (the training set consists of 8760 hourly rows) and the remaining data are used for testing. It is obvious that the testing period differs for each target PV based on the total number of available data; $PV_7$ has the longest testing period, consisting of 13172 hourly rows, while $PV_3$ has the shortest testing period, consisting of 910 hourly rows. Finally, the same processing routine is implemented for all PVs with training data of 3, 6 and 9 months keeping the test set the same, in order to investigate the impact of TL with low data availability.

Finally, the experimental application is implemented on Python programming language, interacting with open-source libraries, including NumPy and Pandas, as well as the DL application program interface (API) TensorFlow (source code is available in GitHub: Source Code). ADAM is the selected optimizer based on existing literature, while the learning rate is set to 0.001. The developed stacked LSTM model is composed of three fully connected intermediate layers (first layer includes 24 neurons, second layer includes 48 neurons and third layer includes 96 neurons) followed by the output layer. The number of epochs is set to 100 and the batch size is set to 128 by the trial-and-error method.

### Transfer learning

**Figure 2:**
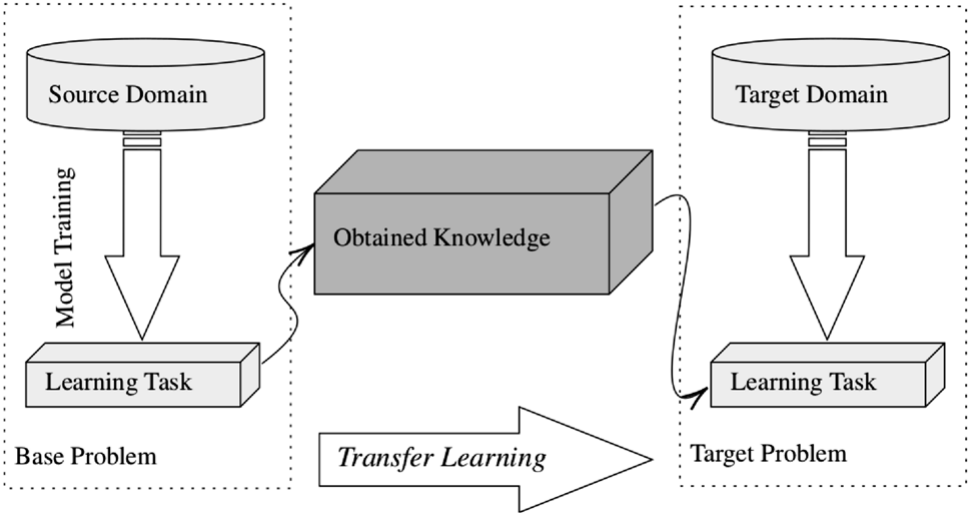
The transfer learning process.

TL is a technique that focuses on exploiting knowledge gained while solving one problem in order to solve a different problem with similar characteristics. The general concept of TL is transferring the expertise of a model from the source domain to the target domain, relaxing the hypothesis that the data of these two problems must be independent and identically distributed^36^. TL provides numerous advantages, namely reduced training time^37^, improved NN performance and, more importantly, the opportunity to achieve high accuracy with limited amount of data^38^. The general framework of the TL process is presented in Fig. 2.

According to the formal definition of TL proposed by Pan and Yang^39^: “Given a source domain ${\displaystyle {{\mathscr {D}}}_{S}}$ and learning task ${\displaystyle {\mathscr {T}}_{S}}$, a target domain ${\displaystyle {{\mathscr {D}}}_{T}}$ and learning task ${\displaystyle {{\mathscr {T}}}_{T}}$, where ${\displaystyle {{\mathscr {D}}}_{S}\ne {{\mathscr {D}}}_{T}}$, or ${\displaystyle {{\mathscr {T}}}_{S}\ne {{\mathscr {T}}}_{T}}$, TL aims to help improve the learning of the target predictive function ${\displaystyle f_{T}(\cdot )}$ in ${\displaystyle {\mathscr {D}}_{T}}$ using the knowledge in ${\displaystyle {\mathscr {D}}_{S}}$ and ${\displaystyle {{\mathscr {T}}}_{S}}$”. This definition is better understood by defining the concepts of domain and task. A domain ${\displaystyle {{\mathscr {D}}}}$ consists of: a feature space ${\displaystyle {{\mathscr {X}}}}$ and a marginal probability distribution ${\displaystyle P(X)}$, where ${\displaystyle X=\{x_{1},...,x_{n}\}\in {{\mathscr {X}}}}$. The feature space can be defined as a collection of features related to specific properties of the data which are given as input to the model. Given a specific domain, ${\displaystyle {\mathscr {D}}=\{{{\mathscr {X}}},P(X)\}}$, a task consists of two components: a label space ${\displaystyle {{\mathscr {Y}}}}$ and an objective predictive function ${\displaystyle f:{{\mathscr {X}}}\rightarrow {{\mathscr {Y}}}}$. The objective predictive function aims to predict the label ${\displaystyle f(x)}$ of each new instance ${\displaystyle x}$.

Finally, according to Lu et al.^40^ there are three main categories of TL methods. Inductive TL assumes that the learning task in the target domain is different from the learning task in the source domain. Unsupervised TL, also assumes that the learning task in the target domain is different from the learning task in the source domain, but focuses only on unsupervised problems such as clustering and density estimation. Finally, transductive TL assumes that the learning tasks are the same in both domains, while the source and target domains are different, but related. The proposed TL approach for the problem of PV production forecasting belongs to the field of transductive TL, because the source and target tasks are the same (hourly PV prediction), while the source and target domains are different in terms of location, nominal capacity and weather conditions.

### The long short-term memory model

One of the most suitable models for the application of TL in the PV production forecasting problem is the LSTM model^41^. This is mainly due to the fact that the functionality of the LSTM depends on weight updating between the neurons of the deep learning model, allowing the creation of pre-trained models. Thus, it facilitates pre-training the model on the baseline PV in order to utilise the saved weights of the pre-trained model and apply TL on the target PV. The same applies for other NNs, but LSTM networks have shown the best performance, and the interest in PV power prediction using variations of LSTM networks has been continuously increasing over the past few years^42^.

The LSTM is a RNN architecture with the innate ability of capturing long term dependencies in sequence prediction problems^43^. The purpose of the LSTM development has been the vanishing gradient problem, which can be described as the exponential increase (or decrease) of the backpropagated error signal as a function of the distance from the final layer, resulting in models which are unstable and incapable of efficient learning^44^. The LSTM uses an additive gradient structure which incorporates direct access to a forget gate enabling the network to stimulate desired behaviour from the error gradient^45^.

**Figure 3:**
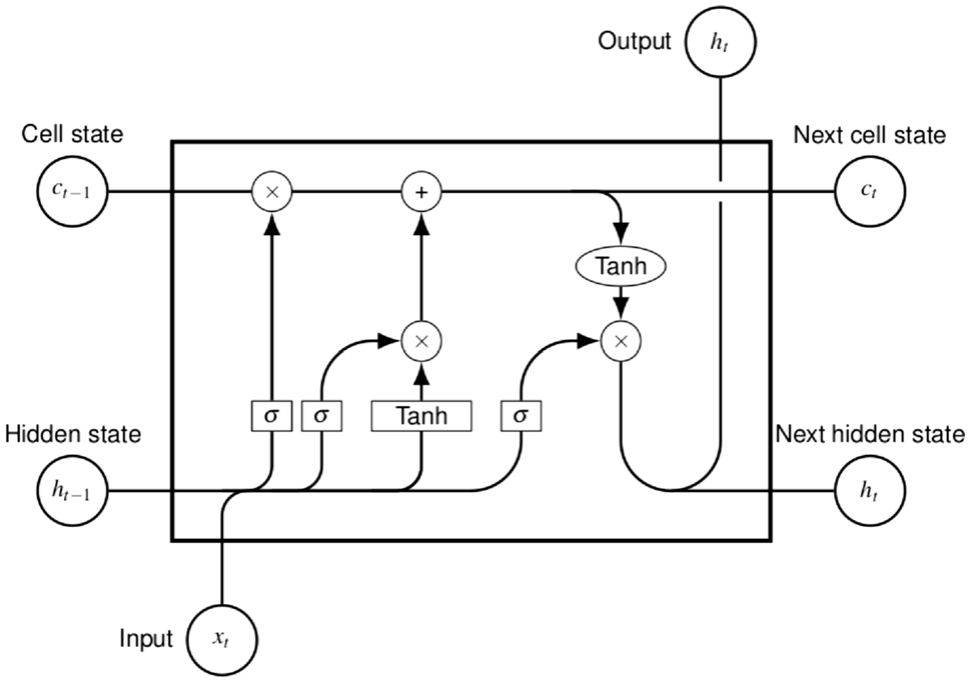
The long short-term memory (LSTM) model architecture.

The selection of LSTM over traditional ML algorithms and feedforward NNs is based on its suitability for holding long term memory, which is essential when facing problems with sequential data with temporal relationship. LSTM is able to represent the dynamic performance of systems, thus being one of the most widely models for dealing with time series problems, such as PV production forecasting^46^. LSTMs provide a significant advantage over other methods, as they are able to detect linear relationships between nonlinear data. Such relationships may appear in the PV production forecasting problem, between power output and meteorological data. In this respect, the LSTM can benefit from features and detect relationships and patterns that other models would not be able to find. Furthermore, LSTM has been exploited for several energy-related time series problems, including residential energy consumption predictions^47,48^, and natural gas demand forecasting^49^. The architecture of the LSTM cell is illustrated in Fig. 3.

The presentation of the LSTM architecture follows the works of Graves^50^ and Olah^51^. The standard LSTM cell includes four NN layers, differing from common RNN architectures which include a single layer. Each line in Fig. 3 represents a vector to which several pointwise operation and NN layers are performed. The LSTM cell receives three inputs and produces two outputs. The inputs, passed in vector form, are the following: the current input $x_{t}$, the previous hidden state $h_{t-1}$ and the previous cell state $c_{t-1}$. The outputs of LSTM are the cell state and the hidden state. On the one hand, the cell state (depicted by the horizontal line at the top) encapsulates the long term memory capability of processing information of more distant events. On the other hand, the hidden state transfers information from immediately previous events and it is overwritten at every step. The core functionalities of the LSTM cell are implemented through its three gates: the forget gate, the input gate and the output gate.

1. The forget gate is the first block represented in the LSTM architecture. The forget gate determines which part of the information must be retained or discarded. The inputs of this gate are the previous hidden state $h_{t-1}$ and the the current input $x_{t}$. These inputs are passed through the sigmoid function $\sigma _{g}$ which results in output values between 0, which denotes that no information passes through, and 1, which denotes that all information passes through. The forget gate’s activation vector $f_t$ is given by the following equation.
  1 $$\begin{aligned} f_{t} =\, \sigma _{g}(W_{f}x_{t}+U_{f}h_{t-1}+b_{f}) \end{aligned}$$
2. The input gate serves as an input to update the cell status. The input gate’s functionality is performed in two parts. Firstly, the previous hidden state and the current input are passed into the second sigmoid function $\sigma _{g}$. Secondly, the same inputs are passed into the hyperbolic tangent function $\sigma _{c}$ in order to regulate the network. Finally, the cell state vector $c_{t}$ is the result of the element-wise product of the cell input activation vector ${\tilde{c}}_{t}$ and the update gate’s activation vector $i_{t}$. The input gate’s activation functions are given by the following equations.
  2 $$\begin{aligned} {\tilde{c}}_{t}=  \sigma _{c} (W_{c}x_{t}+U_{c}h_{t-1}+b_{c}) \end{aligned}$$
  3 $$\begin{aligned} i_{t}=  \sigma _{g}(W_{i}x_{t}+U_{i}h_{t-1}+b_{i}) \end{aligned}$$
  4 $$\begin{aligned} c_{t}=  f_{t}\circ c_{t-1}+i_{t}\circ {\tilde{c}}_{t} \end{aligned}$$
3. Finally, the output gate determines the next hidden state $h_{t}$. The hidden state includes information on previous inputs and it is utilized for prediction. The previous hidden state $h_{t-1}$ and the current input $x_{t}$ are passed into the third sigmoid function $\sigma _{g}$. Then, the modified cell state is passed to the hyperbolic tangent function $\sigma _{h}$. These outputs are multiplied element-wise allowing the network to determine which information the hidden state should carry.
  5 $$\begin{aligned} o_{t}=  \sigma _{g}(W_{o}x_{t}+U_{o}h_{t-1}+b_{o}) \end{aligned}$$
  6 $$\begin{aligned} h_{t}=  o_{t}\circ \sigma _{h}(c_{t}) \end{aligned}$$

The parameters ${\displaystyle W\in {\mathbb {R}} ^{h\times d}}$, ${\displaystyle U\in {\mathbb {R}} ^{h\times h}}$ and ${\displaystyle b\in {\mathbb {R}} ^{h}}$ represent weight matrices and bias vector parameters respectively, which are learned during the training process.

### Proposed architectures

The high accuracy achieved by NNs in a variety of challenging forecasting problems is attributed to their complex architecture. Deep NNs incorporate the concept of hierarchy due to the connection of multiple layers of neurons. Each layer is responsible for solving a small task of the main problem and its output is transferred to the next layer^52^. The solution to the problem is produced by the last layer of the network. The intermediate layers of deep NNs are called hidden layers. The main idea of introducing hidden layers to the architecture is that each hidden layer generates more advanced representations of the problem leading to higher abstraction levels. Thus, deep NNs can represent any non-linear function with relatively fewer neurons than a single-layer network. DL assumes that a hierarchical model with many layers is exponentially more efficient at approximating some functions than a more shallow one^53^.

This approach can also be applied to LSTMs. The original LSTM model is composed of one single layer which receives the input data and passes the output signal to a single feed-forward output layer. However, in this study an alternative architecture is proposed, which involves multiple hidden layers of multiple LSTM units followed by a feed-forward output layer. Each layer provides a sequence output to the next layer, rather than a single value output. This architecture is called stacked LSTM network, and it has been introduced by Graves et al.^54^ in their application of LSTMs to speech recognition. Proportional to simple feed-forward networks, stacked LSTM networks result in deeper models with higher levels of approximation accuracy. Moreover, due to the fact that LSTMs are used with sequence data (their hidden state is a function of all previous hidden states), deeper architectures lead also to deeper level of abstraction of the input data over time providing a representation of the task at different timescales^55,56^.

TL is exploited through the process of reusing the weights of a model which has been trained on the source domain data to fine-tune a new model based on the target domain data. The pre-trained model is referred to as the base model, while each new model in the target domain is referred to as TL model. The weights of each layer of the base model can be processed differently in order to provide better performance of the TL model in the target domain, using the following approaches: (a) keep the weights of the layer fixed, (b) fine-tune the weights of the layer based on the target domain data and (c) train the weights of the layer from scratch based on the target domain data.

In this paper, three TL strategies are developed and compared in terms of forecasting accuracy for the problem of PV production forecasting.

- *TL Strategy 1:* In the first strategy, the weights of the initial layers are frozen and the only trainable weights are the weights of the last hidden layer. This strategy is known as weight freezing and it is widely used in order to extract features from the source domain and carry them to the target domain. This is a widely used scheme when treating images, where the first layers are used as feature extraction layers and the last layers are used to adapt to new data.
- *TL Strategy 2:* In the second strategy, the base model is used as a weight initialization scheme for the TL model. The weights of all layers of the TL model are initialized based on data from the source domain and they are fine-tuned based on data from the target domain. This approach is extensively used with problems where there is an abundance of data in the source domain, but a scarcity of data in the target domain. However, a high degree of similarity between the source and the target domain is a necessary condition.
- *TL Strategy 3:* In the third strategy, the initial layers of the TL model are frozen and the last layer is trained from scratch, popping the last layer of the base model and adding a new layer after the frozen layers. This approach is similar to the first one, but it differs in the fact that the weights of the last layer are not initialized based on data from the source domain. Thus, the TL model serves as a feature extraction mechanism because of the frozen layers, but it can also be fine-tuned to the target domain because of the random initialization of the last layer’s weights.

## Results

The results of this study are presented in three categories, namely; the forecasting performance of the baseline stacked LSTM model, the TL models performance results compared to the conventional model in the target domain and the results of applying TL with different volume of available data, respectively. By the term conventional model we refer to the LSTM model in which no TL has been applied; in this context, the conventional LSTM model is solely based on training with data from the target PV.

### Baseline model performance

The stacked LSTM model has the following lag features: (a) Power output measured value, (b) air temperature, (c) global horizontal irradiance, (d) humidity, (e) month of the year (in the form of one-hot encoding) and (f) hour of the day (in the form of sine/cosine transformation). The above-mentioned features are fed into the LSTM model in the format of “5 inputs - 1 output” of hourly data. More specifically, a point value for each feature is fed into the model for the last five hours and the PV power output for the next hour is predicted (one-hour ahead power output forecast).

Ensuring an accurate base model is a prerequisite for achieving accurate predictions in the target domain. In this context, the performance of the LSTM model for the base PV is evaluated with the following procedure: The base PV dataset is split into train set and evaluation set using a 80-20 split, keeping the first 80% as training and the remaining 20% as testing (17563 observations for the training process and 4391 observations for evaluation purposes) and the LSTM model is trained on the training set. The accuracy of the model is evaluated by computing the root mean squared error (RMSE) and the mean absolute error (MAE) of the respective forecasts across the evaluation period considered, as well as the coefficient of determination $R^2$ between the forecasts and the real values, as follows:

7 $$\begin{aligned} RMSE= & {} \sqrt{ \frac{1}{n} \displaystyle \sum _{t=1}^{n} ({y_t-\hat{y_t}})^2 } \end{aligned}$$

8 $$\begin{aligned} MAE= & {} \frac{1}{n} \displaystyle \sum _{t=1}^{n} | {y_t-\hat{y_t}|} \end{aligned}$$

9 $$\begin{aligned} R^2= & {} 1 - \frac{ \displaystyle \sum _{t=1}^{n} ({y_t-\hat{y_t}})^2}{\displaystyle \sum _{t=1}^{n} ({y_t-{\bar{y}}})^2 } \end{aligned}$$

where $y_t$ is the real value of the solar production time series at hourly interval *t* of the evaluation period, $\hat{y_t}$ is the produced forecast of the model and ${\bar{y}}$ is the average of the real values. Apart from these error metrics, two additional metrics are calculated in order to make the model evaluation more complete: the Mean Bias Error (MBE) and the normalized root mean squared error (NRMSE). The MBE represents the systematic error of a forecasting model to under or overforecast, while the NRMSE is suitable for the comparison between models of different scales connecting the RMSE value with the observed range of the variable. These two metrics are calculated as follows:

10 $$\begin{aligned} MBE= & {} \frac{1}{n} \displaystyle \sum _{t=1}^{n} ( {y_t-\hat{y_t})} \end{aligned}$$

11 $$\begin{aligned} NRMSE= & {} \frac{RMSE}{{\bar{y}}} \end{aligned}$$

The model achieves high accuracy, managing to efficiently capture the daily patterns of the most important variables, as reflected by the utilized metrics ($MAE = 0.467$, $RMSE = 0.992$, $MBE = -\,0.097$, $nRMSE = 0.301$, $R^2 = 96.254\%$). However, even these five error metrics are not enough to sufficiently illustrate the capabilities of the proposed model in comparison with other models in different geographical locations. According to Yang et al.^57,58^, the accuracy of solar forecasting models (in general, the term “solar forecasting” may refer to either solar irradiance forecasting or solar power forecasting; throughout this study the term refers to solar power forecasting) must be inter-comparable across different locations and different time periods through a common metric which is the forecast skill index. The forecast skill index is based on the comparison of the proposed model to a reference model on a specific error metric. However, two issues arise: What reference model and which error metric must be used? The most common reference model to standardize the verification of solar forecasting models is the persistence model. More specifically, the utilization of a smart persistence model as a reference model is highly recommended, rather that using the naive (or simple) persistence model^59^. Regarding the optimal error metric, the RMSE is the most suitable metric in the case of solar power production, as a metric that is appropriate for capturing large errors^57^. Thus, the formula of the forecast skill index is the following:

12 $$\begin{aligned} skill = 1 - \frac{RMSE_{proposed}}{RMSE_{reference}} \end{aligned}$$

where $RMSE_{proposed}$ is the RMSE value of the developed LSTM model and $RMSE_{reference}$ is the RMSE value of the smart persistence model.

**Figure 4:**
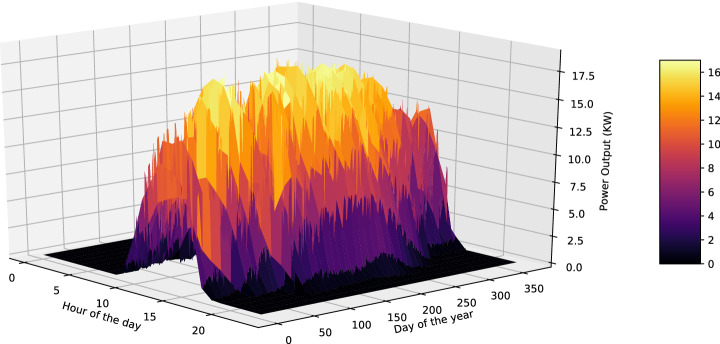
The power output expected under clear sky conditions for the base PV as a function of the hour of the day (ranging between 0 and 23) and the day of the year (ranging between 0 and 255).

The last question that arises concerns the selection of the smart persistence model in the case of solar power forecasting. For solar irradiance forecasting problems, the smart persistence model derives from integrating clear sky conditions to the reference model^59^. The same also applies to PV power forecasts, where several smart persistence models have been proposed^60^. More specifically, a clear sky index has been proposed by Engerer and Mills in case that the characteristics of the PV panel are known^61^, while another PV smart persistence model based on scaling global horizontal irradiance to PV production value has been presented by Huertas and Centeno^62^. In this study, the definition of Pedro and Coimbra is adopted, which is based on estimating the expected power output under clear-sky conditions^63^. The formula of the adopted smart persistence model is described by the following equation:

13 $$\begin{aligned} {\hat{y}}(t+\Delta t) = {\left\{ \begin{array}{ll} y_{c-s}(t+\Delta t), &{} if\ y_{c-s}(t) = 0 \\ y_{c-s}(t+\Delta t) \frac{y(t)}{y_{c-s}(t)} &{} otherwise \end{array}\right. } \end{aligned}$$

where *y*(*t*) is the measured power output and $y_{c-s}(t)$ represents the expected power output under clear-sky conditions. The purpose of this model is to decompose power output, indicating that a fraction of the power output relative to the clear-sky conditions remains the same between short time intervals. Moreover, at night conditions the forecast of the smart persistence model is considered equal to the clear sky power output. The approximated function for the clear-sky model can be created by averaging past power output values depending on the hour of the day (between 0 and 23) and the day of the year (between 0 and 255). The second step involves creating the smooth surface that envelops the above-mentioned function^63^. The power output expected under clear sky conditions for the base PV ($PV_1$) as a function of the hour of the day and the day of the year for the baseline model is presented in Fig. 4.

The smart persistence model performance is reflected by the following error metrics: $MAE = 0.582$, $RMSE = 1.274$, $MBE = 0.029$, $nRMSE = 0.387$, $R^2 = 93.811\%$. Although the smart persistence model shows quite good performance in comparison with the naive persistence model ($RMSE_{Naive} = 1.985$, $MAE_{Naive} = 1.110$), it is evident that the LSTM significantly outperforms the smart persistence model. This is also highlighted through the forecast skill index of the LSTM model which is equal to 0.221. A positive forecast skill index indicates that the proposed model outperforms the smart persistence model, while a negative one shows that the smart persistence model performs better.

**Figure 5:**
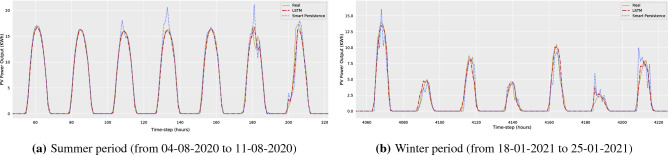
Example illustrating how the solar power forecasting model performs in comparison with the smart persistence model. The horizontal axis indicates the hourly time-step of the evaluation period, while the vertical axis shows the solar power production. The example refers to a randomly selected summer week (between hours 54 and 222 of the validation set, corresponding to 04-08-2020 and 11-08-2020) and a randomly selected winter week (between hours 4060 and 4228 of the validation set, corresponding to 18-01-2021 and 25-01-2021) of the evaluation period.

Finally, Fig. 5 depicts the results of the forecasting models (LSTM baseline model and smart persistence model) for two different periods. It can be concluded that the model manages to capture seasonality, trends and weather-related variations both in summer and winter periods, and thus offer significantly better forecasts compared to the smart persistence model.

### Transfer learning methods

The TL models are equipped with exactly the same characteristics as the baseline model, using the baseline pre-trained model to solve exactly the same problem, with the same features and the same expected output, in a different PV plant. Therefore, the features of the TL models are: (a) Power output measured value, (b) air temperature, (c) global horizontal irradiance, (d) humidity, (e) month of the year (one-hot encoding) and (f) hour of the day (sine/cosine transformation) and the model output is a one-hour ahead forecast of the PV power output.

The validation process of the proposed TL strategies is implemented in 6 PV plants, with different nominal and peak capacity, located in 4 cities in Portugal. Four architectures are compared, including the presented TL strategies, as well as a conventional model where no TL has been applied. For the TL models, a pre-training is applied on the whole dataset of the base PV (30 months of data). Then, the four models are trained using one year of data (8760 h) and they are tested in the rest of the dataset. For each PV plant the size of the test dataset is different depending on data availability, as presented in Table 1. The models’ accuracy is evaluated based on their performance on the evaluation data using *RMSE*, *MBE*, *MAE*, *NRMSE* and $R^2$.

20 training repetitions are performed for each model, in order to eradicate randomness. This number of repetitions is generally proposed in the literature. The forecasting performance for all models is presented in Table 2, where the average values of RMSE, MBE, MAE, NRMSE and $R^2$ are reported, providing some very useful insights.

Firstly, it is worth mentioning that all LSTM models perform better than the smart persistence model in terms of RMSE. This fact illustrates the suitability of the selected model and the selected features for this problem. The only case that the LSTM performs worse than the smart persistence model is for the conventional LSTM of $PV_2$. Even in this case the three TL models have lower error indexes than the smart persistence one. The forecast skill index varies between $-\,0.15$ (it is negative in the case of $PV_2$) and 0.48 for the conventional model, while it varies between 0.28 and 0.56 for the TL strategies. The average percentage increase of the forecasting skill index between the conventional and the TL models is $16.3\%$. Finally, the MBE index shows that none of the developed models shows any indication of bias.

**Table 2:** Average forecasting performance (accuracy) of the smart persistence model and the stacked LSTM models with and without TL.

PV	Metric	Without TL	Smart Persistence	TL Strategy 1	TL Strategy 2	TL Strategy 3
$PV_2$	*RMSE* (KWh)	3.77	3.27	2.34	2.37	2.36
	*MBE* (KWh)	$-$ 0.25	0.17	0.16	0.06	0.05
	*MAE* (KWh)	1.81	1.38	1.11	1.11	1.12
	*nRMSE* (%)	53.14	46.18	33.06	33.38	33.28
	$R^2$ (%)	87.74	91.37	95.57	95.49	95.51
$PV_3$	*RMSE*	11.39	17.32	10.59	11.35	11.48
	*MBE*	0.83	0.02	0.79	0.81	0.80
	*MAE*	6.01	6.49	5.29	5.94	5.85
	*nRMSE*	40.78	62.15	37.94	40.63	41.13
	$R^2$	93.60	85.27	94.49	93.68	93.53
$PV_4$	*RMSE*	1.65	2.69	1.48	1.46	1.46
	*MBE*	$-$ 0.01	$-$ 0.82	0.11	0.06	0.07
	*MAE*	0.81	1.32	0.70	0.69	0.70
	*nRMSE*	35.57	57.84	31.94	31.42	31.51
	$R^2$	94.46	85.40	95.55	95.70	95.68
$PV_5$	*RMSE*	2.66	5.27	2.31	2.31	2.31
	*MBE*	$-$ 0.08	0.35	$-$ 0.04	$-$ 0.11	$-$ 0.16
	*MAE*	1.37	1.52	1.13	1.15	1.15
	*nRMSE*	31.68	62.75	27.56	27.46	27.54
	$R^2$	95.17	81.51	96.43	96.46	96.44
$PV_6$	*RMSE*	6.75	8.59	5.65	5.50	5.51
	*MBE*	1.35	$-$ 0.04	0.94	0.43	0.56
	*MAE*	3.54	4.25	2.74	2.79	2.77
	*nRMSE*	40.92	52.11	34.23	33.37	33.40
	$R^2$	92.72	88.58	95.07	95.33	95.31
$PV_7$	*RMSE*	3.64	4.59	2.47	2.40	2.41
	*MBE*	$-$ 0.16	0.15	0.24	$-$ 0.02	0.03
	*MAE*	1.75	1.64	1.15	1.11	1.12
	*nRMSE*	40.87	51.49	27.78	26.97	27.08
	$R^2$	92.23	88.19	96.56	96.76	96.74

*RMSE*, *MBE* and *MAE* are measured in KWh, while for $R^2$ and *nRMSE* the percentage is given for each model

**Figure 6:**
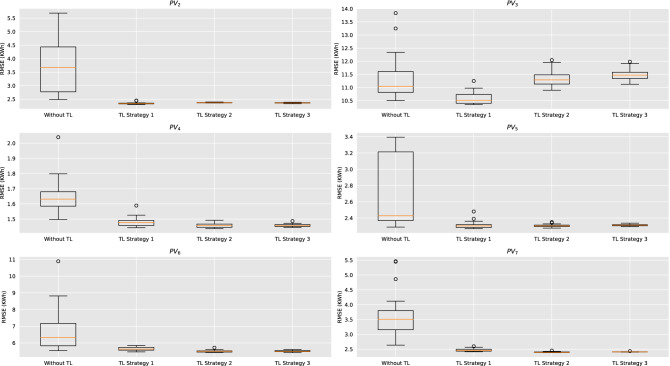
Boxplot that summarizes the performance of the four stacked LSTM models for the six target PV plants based on the RMSE. Base stands for the model that no TL has been applied, while TL1, TL2, TL3 stand for the three TL strategies, respectively.

Regarding the comparison between the conventional LSTM and the three TL models, the impact of TL is evident as TL strategies have better accuracy than the conventional one for all six PVs . The boxplots presented in Fig. 6 also show that the conventional LSTM has greater RMSE average value in all target PV plants, while it also demonstrates a bigger variance compared to the TL models. Indeed, the models that are used without TL suffer from high variance, offering considerably different accuracy in each repetition. On the contrary, models trained with the three TL strategies show nearly zero variance, while also achieving more accurate, non-biased forecasts. A remarkable point is that for $PV_3$, where the evaluation period is only 38 days (910 hourly point forecasts), the three TL models do not seem to outperform the conventional model in the extent that they do for the other PV plants. This is due to the fact that the evaluation takes place solely on March (winter period) where the problem is more complex as weather patterns are often disturbed, while another sign illustrating the forecasting difficulty in $PV_3$ is that neither the smart persistence model is able to make better forecasts.

### Data availability impact

As mentioned in the introductory section, one calendar year of data is the minimum time interval for a model to be sufficiently trained, in order to incorporate all seasonal and weather patterns of the problem. Also, the presented results indicate that TL models can perform better than conventional models considering a scenario where one year of training data is available, while conventional models are still better than reference smart persistence ones. However, the application of TL offers the possibility to obtain reliable and accurate predictive models, even when the available training data for the target domain are less than one year. In this context, the proposed architectures are compared on the target PVs for different training periods, namely 3 months, 6 months and 9 months of available data. It must be noted that, although the training period has changed, the testing period has been kept the same for comparison purposes between the different scenarios.

**Figure 7:**
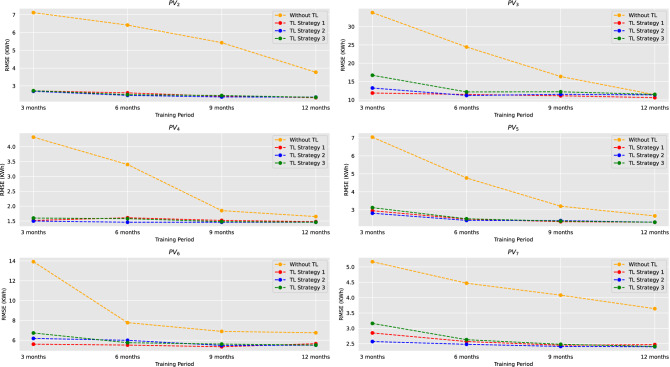
Comparative performance of the four models based on RMSE index for 3 month, 6 month, 9 month and 12 month training period for the six target domain PVs.

Figure 7 presents the RMSE index of the four models in the four aforementioned scenarios of different training periods. Results indicate that the TL models are more robust considering different volumes of training data and that their performance slightly improves when more data are available. This can be contributed to their anterior training on the base PV over 3 years of hourly data. On the other hand, the impact of data scarcity is apparent for the conventional LSTM model, which radically improves when the training period increases and identifies new seasonal and weather patters. It is worth mentioning that none of the 3-month trained conventional models outperforms the smart persistence model, while only three 6-month trained conventional models manage to achieve better accuracy compared to the smart persistence one. The same does not apply for the 3-month trained TL models, which have lower RMSE compared to both the conventional LSTM and the smart persistence model.

Finally, the difference in terms of RMSE between the conventional model and the best-performing TL model decreases as more training data are becoming available. This is evident in all six target PVs. For example, the difference in terms of RMSE in $PV_5$ is limited from $150.5\%$ (3-month training models) to $15.1\%$, about 10 times lower. Same decrease patterns are also identified in the other five PVs, further highlighting the importance of TL, especially when less than one calendar year of data is available.

## Discussion

Collecting sufficient data from recently installed solar plants is a long process. The urgent need for accurate PV production forecasts has led to the idea of exploiting solar plants with sufficient data to provide accurate forecasts for recently installed ones. In this paper, the purpose of the research is to determine whether TL can be efficiently employed to provide PV production forecasts for solar plants with limited data size. Three TL strategies based on a stacked LSTM architecture are developed and compared to a non-TL approach. The presented methodology is tested in power plants in different cities and with different nominal capacities. The findings of the experimental application indicate that all three TL strategies significantly outperform the non-TL approach in terms of forecasting accuracy, evaluated by several error indexes.

Moreover, the models are compared with a smart persistence model based on the clear-sky power output. The models which are trained with the three TL strategies significantly outperform the reference model, having a forecast skill score between 0.28 and 0.56 considered satisfying by the existing literature. On the opposite side, the non-TL LSTM model shows limited forecasting accuracy, quantified by an average decrease of $16.3\%$in the forecast skill index. The aforementioned results correspond to the simulation scenario in which one calendar year of data is available. Results of additional experiments using varying volumes of training data suggest that the less data available, the greater the gap between TL strategies and the non-TL approach, further necessitating the use of TL. Especially in the scenario that 3 months of data are available for training, the gap between the conventional model and the TL ones significantly increases, while the conventional LSTM fails to outperform even the reference model.

Last but not least, one of the most significant parameters in the concept of TL is the replicability of the presented TL strategies. In order to assess this aspect, the experimental application has been performed on three PV panels located in the same city as the base PV and on three PV panels located in different cities. This enables comparison between the two groups in terms of forecasting accuracy. However, the fact that these PV panels are located in different cities and have different nominal power indicates that the comparison must take place on the forecast skill index, to be in alignment with the proposed verification guidelines for deterministic solar forecasts. Thus, the average forecast skill index for PV panels in the same city is 0.4, while the corresponding value for PV panels in different cities is 0.43. This is undoubtedly a sign that the forecasting accuracy of the models is not affected by the geographical distance between the base and the target PV.

This study is the first step towards enhancing our understanding of the impact of TL on solar plant power prediction. Future work will concentrate on assessing the impact of the base model’s training data volume, investigating whether training base models with more data or with data from different solar plants could further improve forecasting accuracy. This could result in the evolution of cross-stakeholder models and data sharing among energy communities, with the aim to promote inclusiveness in smart cities environments. Further studies, which take geographical characteristics differences between the base and the target domain into account (i.e., altitude, solar plant orientation), will also need to be performed. Finally, the prospect of being able to use TL for solar plants power forecasting, serves as a continuous incentive for future research on transferring knowledge in similar problems, such as cross-building energy forecasting, wind power prediction, and hydraulic plant generation forecasting, among others.

## Data Availability

The meteorological data included in this study are available within the Copernicus Atmosphere Data Store - via CAMS Solar Radiation time series database (https://ads.atmosphere.copernicus.eu) and the Weather Underground (https://www.wunderground.com) website. User registration required (free) for downloading. All power output data analyzed during this study can also be provided upon request.

## Code availability

The deep learning-based pre-trained models for PV power output forecasting and the source code required for reproducing the results of this study are available at https://github.com/ElissaiosSarmas/Transfer-learning-strategies-for-solar-power-forecasting-under-data-scarcity.
